# Clozapine: overview of high-risk drug associations in French psychiatric hospitals. A multicenter survey on a given day

**DOI:** 10.1192/j.eurpsy.2025.619

**Published:** 2025-08-26

**Authors:** M. Bordes, R. Klein, E. Queuille

**Affiliations:** 1 Ferrepsy, Toulouse; 2 CH Charles Perrens, Bordeaux; 3 Association du réseau PIC, Armentieres, France

## Abstract

**Introduction:**

Clozapine is the reference treatment for resistant schizophrenia. Its pharmacokinetic characteristics (metabolism by cytochromes CYP1A2, CYP2C19, and CYP3A4, among others) as well as its pharmacodynamic properties are the source of numerous high-risk drug interactions. According to the French marketing authorization, clozapine is contraindicated with bone marrow depressants, and associations with benzodiazepines, omeprazole, fluvoxamine, and lithium require specific precautions.

**Objectives:**

Collaboration between a national multi-professional network operating in various public or private mental health hospitals (the PIC network) and a regional psychiatric research federation (FERREPSY Occitanie) enabled the implementation of a study describing the prevalence of high-risk drug associations in a large panel of French psychiatric hospitals

**Methods:**

An observational cross-sectional study was conducted in December 2023 across 30 centers that are members of the PIC network and/or FERREPSY.

**Results:**

The medical records of 795 patients were analyzed by hospital pharmacists from the participating centers. Several high-risk associations with clozapine were identified. In 1.5% of cases, clozapine was associated with carbamazepine, in 1.1% of cases with omeprazole, and in 3% of cases with fluvoxamine. More frequently, associations with lithium salts were found in 15.6% of patients and with benzodiazepines in 68.3% of patients.

**Conclusions:**

This study provided an overview of high-risk co-prescriptions with clozapine in French psychiatric institutions. It highlights a high prevalence of certain high-risk associations, which underscore the discrepancy between clinical practices and health agency recommendations.

**Disclosure of Interest:**

None Declared

